# FADS3 is a Δ14Z sphingoid base desaturase that contributes to gender differences in the human plasma sphingolipidome

**DOI:** 10.1074/jbc.AC119.011883

**Published:** 2019-12-20

**Authors:** Gergely Karsai, Museer Lone, Zoltán Kutalik, J. Thomas Brenna, Hongde Li, Duojia Pan, Arnold von Eckardstein, Thorsten Hornemann

**Affiliations:** ‡ Institute for Clinical Chemistry, University Hospital and University Zurich, 8091 Zürich, Switzerland; § University Center for Primary Care and Public Health, University of Lausanne, 1010 Lausanne, Switzerland; ¶ Swiss Institute of Bioinformatics, 1015 Lausanne, Switzerland; ‖ Dell Pediatric Research Institute, Departments of Chemistry, Pediatrics, and Nutrition, University of Texas, Austin, Texas 78723; ** Department of Physiology, University of Texas Southwestern Medical Center, Dallas, Texas 75390

**Keywords:** sphingolipid, lipid metabolism, human genetics, single-nucleotide polymorphism (SNP), molecular cell biology, ceramide, genomics, fatty acid desaturase 3 (FADS3), GWAS

## Abstract

Sphingolipids (SLs) are structurally diverse lipids that are defined by the presence of a long-chain base (LCB) backbone. Typically, LCBs contain a single Δ4E double bond (DB) (mostly d18:1), whereas the dienic LCB sphingadienine (d18:2) contains a second DB at the Δ14Z position. The enzyme introducing the Δ14Z DB is unknown. We analyzed the LCB plasma profile in a gender-, age-, and BMI-matched subgroup of the CoLaus cohort (*n* = 658). Sphingadienine levels showed a significant association with gender, being on average ∼30% higher in females. A genome-wide association study (GWAS) revealed variants in the fatty acid desaturase 3 (*FADS3*) gene to be significantly associated with the plasma d18:2/d18:1 ratio (*p* = −log 7.9). Metabolic labeling assays, FADS3 overexpression and knockdown approaches, and plasma LCB profiling in FADS3-deficient mice confirmed that FADS3 is a *bona fide* LCB desaturase and required for the introduction of the Δ14Z double bond. Moreover, we showed that FADS3 is required for the conversion of the atypical cytotoxic 1-deoxysphinganine (1-deoxySA, m18:0) to 1-deoxysphingosine (1-deoxySO, m18:1). HEK293 cells overexpressing FADS3 were more resistant to m18:0 toxicity than WT cells. In summary, using a combination of metabolic profiling and GWAS, we identified FADS3 to be essential for forming Δ14Z DB containing LCBs, such as d18:2 and m18:1. Our results unravel FADS3 as a Δ14Z LCB desaturase, thereby disclosing the last missing enzyme of the SL *de novo* synthesis pathway.

## Introduction

Sphingolipids (SLs)^2^ are a diverse class of lipids that share a long-chain base (LCB) backbone as a common structural element. Forming an LCB is the first and rate-limiting reaction in the *de novo* synthesis of SL (Fig. S1). During SL synthesis, LCBs are usually *N*-acylated to a fatty acid (FA) of variable length (C_16_–C_26_) forming ceramides and conjugated to variable headgroup structures forming complex SLs. LCBs are synthesized at the endoplasmic reticulum by the enzyme serine palmitoyltransferase. The most abundant LCB in mammals is the 18-carbon dihydroxy-amino-alkane sphingosine (d18:1) that is formed by the conjugation of l-serine with palmitoyl-CoA. Serine palmitoyltransferase, however, can metabolize a variety of other acyl-CoA in the range of C_14_–C_18_ and use alanine and glycine as alternative substrates. This forms a broad variety of LCBs, which differ by structure, function, and metabolism (1).

1-DeoxySLs are atypical SLs, which are generated from alanine instead of serine and involved in a variety of pathological conditions, including the rare hereditary sensory neuropathy type 1 (HSAN1) (2). Typically, LCBs present with a canonical Δ4E double bond (DB). However, the -dienic sphingoid base sphingadienine (d18:2) that is formed downstream of sphingosine (d18:1) has an additional DB at the Δ14Z position (3, 4). Recently, we reported that 1-deoxySO (m18:1) contains a Δ14Z DB but lacks the canonical DB at the Δ4E position (5). Whereas the Δ4E DB is introduced by the ceramide desaturase 1 (DEGS1), the enzyme responsible for the introduction of the Δ14Z DB is not known. Here, we identified fatty acid desaturase type 3 (FADS3) as a *bona fide* sphingoid base desaturase introducing a DB in the Δ14Z position and therefore responsible for converting d18:1 into d18:2 and 1-deoxySA (m18:0) into 1-deoxySO (m18:1).

## Results

Human plasma contains different SL classes, of which the most abundant are sphingomyelins (SM), hexosylceramides (HexCer), and ceramides (Cer). These classes are formed on a variety of LCB backbone structures, and variations in their LCB composition are associated with different pathological conditions (6–8). Differences in the LCB profile predict the risk for future diabetes type 2 (T2DM) independent from classical risk factors, such as BMI and blood glucose (9). Males and females have different risk profiles for T2DM. In men, T2DM is more frequently diagnosed at lower age and BMI, whereas obesity is more common in women. We therefore compared the LCB profile in an age- and BMI-matched subgroup of female and male participants of the CoLaus cohort (*n* = 329 each). LDL cholesterol, triglycerides, and fasting glucose were not different between groups (Table 1), whereas total and HDL cholesterol were higher and waist/hip ratio was lower in females (*p* = −log 49.4). In plasma and tissue, the majority of LCBs is *N*-acylated and conjugated to variable headgroups. Nonconjugated LCBs in plasma are minor and less than 1% of the total. The combination of a variable LCB with a variable FA frequently results in the formation of isomeric structures (*e.g.* d18:1/24:0 *versus* d18:0/24:1), which interfere with the LCB analysis by LC-MS. To avoid interference with the *N*-acyl chain, we subjected the extracted lipids to a sequential acid/base hydrolysis (10) to remove the conjugated FA and headgroups. We therefore reported total LCB concentrations without considering previously conjugated *N*-acyl and headgroup structures.

**Table 1:** **Baseline values of clinical parameters and long-chain base levels for each group (significant differences in bold)**

Parameters		Female		Male		-Fold difference	−log(*p*)
		Mean	S.D.	Mean	S.D.		
*n*		329		329			
Age		59.77	9.07	58.31	10.52	1.02	1.24
BMI		28.61	5.03	27.82	4.28	1.03	1.50
**Waist/hip ratio**		**0.86**	**0.07**	**0.94**	**0.06**	**0.91**	**49.39**
**Cholesterol (total)**		**5.94**	**1.09**	**5.63**	**0.94**	**1.06**	**4.08**
**HDL**		**1.66**	**0.40**	**1.38**	**0.31**	**1.20**	**21.24**
LDL		3.59	0.95	3.51	0.82	1.02	0.66
Trig		1.51	0.70	1.63	0.84	0.93	1.37
Glu		6.02	1.67	6.14	1.79	0.98	0.43
**C_16_SO**	**d16:1**	**19.63**	**5.73**	**16.58**	**5.21**	**1.18**	**11.58**
**C_16_SA**	**d16:0**	**0.51**	**0.20**	**0.44**	**0.18**	**1.14**	**4.65**
**C_17_SO**	**d17:1**	**9.52**	**2.63**	**7.91**	**2.28**	**1.20**	**15.45**
C_18_PhytoSO	t18:0	0.15	0.05	0.14	0.04	1.02	0.47
**C_18_SO**	**d18:1**	**97.23**	**19.15**	**88.32**	**16.54**	**1.10**	**9.45**
**C_18_SA**	**d18:0**	**3.51**	**1.08**	**3.12**	**1.00**	**1.12**	**5.48**
**C_18_SAdienine**	**d18:2**	**35.05**	**7.80**	**28.01**	**6.33**	**1.25**	**32.26**
**C_19_SO**	**d19:1**	**2.45**	**0.96**	**2.16**	**0.91**	**1.14**	**4.12**
C_20_SO	d20:1	0.20	0.06	0.19	0.05	1.03	0.57
C_20_SA	d20:0	0.02	0.01	0.02	0.01	1.02	0.28
**1-DeoxySO**	**m18:1**	**0.15**	**0.08**	**0.17**	**0.09**	**0.85**	**4.10**
1-DeoxySA	m18:0	0.08	0.04	0.09	0.04	0.95	0.85

**Figure 1.**
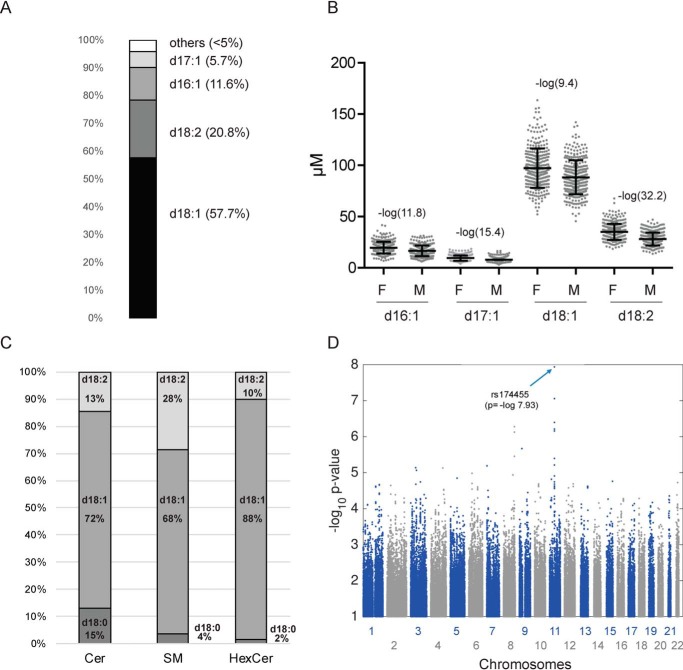
**LCB profiling and genetic associations.** *A* and *B*, long-chain base profile in human plasma. *A*, relative distribution of the most abundant LCBs in human plasma. *B*, differences in the concentration of the major plasma LCBs between females (*F*) and males (*M*). *C*, relative distribution of the LCB species d18:0, d18:1, and d18:2 in Cer, HexCer, and SM in human plasma. The LCBs reflect the sum of Cer (C_16:0_, C_18:0_, C_20:0_, C_22:0_, C_24:0_, and C_24:1_), SM (C_16:0_, C_18:0_, C_20:0_, C_22:0_, C_24:0_, and C_24:1_), and HexCer (C_16:0_, C_22:0_, C_24:0_, and C_24:1_). *D*, Manhattan plot of the GWAS revealed SNP rs174455 on Chromosome 11 to be significantly associated with the d18:1/d18:2 ratio (*p* = −log 7.93). *Error bars*, S.D.

The LCB profiling revealed d18:1 as the most abundant LCB in plasma (57%) followed by d18:2 (21%), d16:1 (5%), and d17:1 (4.2%). The remaining LCB species were less than 5% in total (Fig. 1*A*). Significant gender differences were seen for d18:2 (*p* = −log 32.3) followed by d17:1 (*p* = −log 15.5) and d16:1 (*p* = −log 11.6) LCBs (Fig. 1*B*). In average, d18:2 was about 30% higher in females, whereas its precursor d18:1 was only 10% increased. This indicates that the d18:1 to d18:2 conversion differs between genders. However, the enzyme responsible for this conversion was not known.

To identify the responsible Δ14Z desaturase, we used genome-wide SNP data, which were available for all European individuals of the cohort (11). As the enzyme converts d18:1 into d18:2, we hypothesized that genetic variations in the desaturase gene will be reflected by changes in the d18:1/d18:2 ratio. We therefore used this ratio as a metabolic outcome trait for a GWAS on 1100 participants of the CoLaus cohort for whom the LCB profile was already available from an earlier study (9). The analysis revealed a group of adjacent SNPs, showing a significant association with the d18:1/d18:2 ratio. The strongest association was seen for SNP rs174455 (*p* = −log 7.93), which is located in an intronic region of the fatty acid desaturase 3 (FADS3) gene on chromosome 11q12.2–13.1 (Fig. 1*D*). The same region clusters two other desaturases, FADS1 and FADS2, which metabolize polyunsaturated fatty acids (12). In contrast, the function of FADS3 was still elusive. The FADS3 gene spans 17.9 kb of genomic DNA and has the same structure as FADS1 and FADS2, consisting of 12 exons and 11 introns. On the protein level, FADS3 is 52 and 62% homologous to FADS1 and FADS2, respectively.

**Figure 2.**
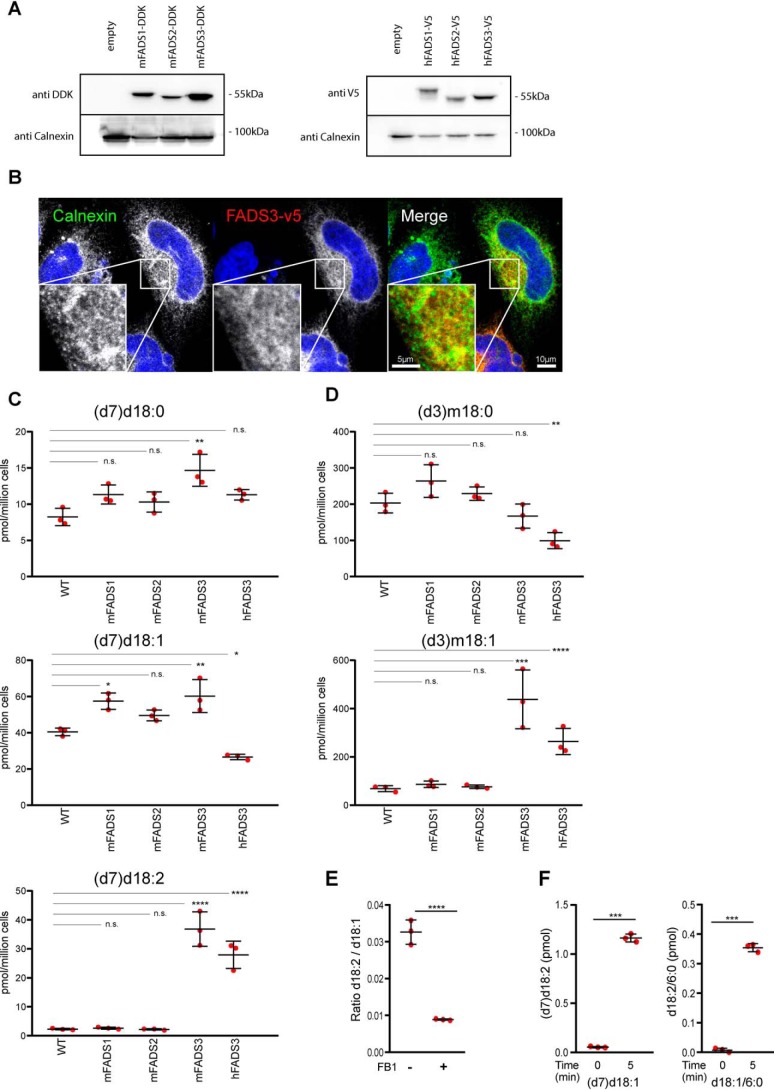
**FADS3 mediates the Δ14 LCB desaturation in mammalian cells.** *A*, Western blotting of HEK293 cells stably expressing mFADS1–3 (Myc-DDK) and hFADS1–3 (V5). *B*, intracellular localization of hFADS3-V5 (*red*) overlaps with the ER marker calnexin (*green*). *Scale bar*, 10 μm. *Insets*, 3-fold magnification. *Scale bar*, 5 μm. *C*, cells stably expressing mFADS1–3 and hFADS3 cultured for 48 h with isotope-labeled (d7)d18:0. The levels of (d7)d18:2 were significantly elevated in mFADS3- and hFADS3-overexpressing cells but not altered in mFADS1 and mFADS2 cells. *D*, FADS1–3–expressing HEK293 cells supplemented with isotope-labeled (d3)m18:0 for 48 h. (d3)m18:1 levels were higher in FADS3-expressing cells compared with WT, mFADS1, and mFADS2 cells. *E*, HEK293 cells supplemented with (d7)d18:1 in the presence or absence of FB1 (35 μm). (d7)d18:2 formation was reduced but still detectable in the presence of FB1, indicating that FADS3 can also metabolize the free LCB. *F*, FADS3 *in vitro* activity with the free LCB ((d7) d18:1) and the *N*-acylated form (d18:1/6:0). The free and the *N*-acylated form were both rapidly converted into (d7)d18:2 and d18:2/6:0, respectively. Data are shown as mean ± S.D. (*error bars*), *n* = 3, paired *t* test; ***, *p* < 0.001; ****, *p* < 0.0001; *n.s.*, not significant.

To test whether FADS3 is a Δ14Z LCB desaturase, we expressed the mouse and human FADS3 cDNA in HEK293 cells. Endogenous d18:2 levels in HEK293 cells were low, indicating a low activity of the putative Δ14Z desaturase. For comparison, we also expressed the cDNA of mFADS1 and mFADS2. Expression levels were comparable for all constructs (Fig. 2*A*). Immune fluorescence microscopy showed an intracellular colocalization of FADS3 with calnexin, indicating that FADS3 is an ER protein (Fig. 2*B*).

Enzyme activity was tested in human and mouse FADS3-expressing cells supplemented either with isotope-labeled (d7)d18:0 or (d3)m18:0. The free LCBs were absorbed by the cells (13) and metabolized to (d7)ceramide and downstream products, such as (d7)SM and (d7)HexCer (Fig. S2*A*). After 48 h, the lipids were extracted and hydrolyzed, and the LCB profile were analyzed by LC-MS. In both mFADS3- and hFADS3-expressing cells, we observed a strong increase in d18:2 (Fig. 2*C* and Fig. S2*B*), which was not seen in mFADS1- or mFADS2-expressing cells. In addition, FADS3-expressing cells showed an increased conversion of m18:0 into m18:1, indicating that the Δ14Z DB in 1-deoxySO is also formed by FADS3 (Fig. 2*D* and Fig. S2*C*). To test whether FADS3 acts on *N*-acylated or free LCBs, we treated cells with fumonisin B1 (FB1). FB1 inhibits ceramide synthase and therefore the *N*-acylation of LCBs. In the presence of FB1, the conversion was about 70% reduced, but a significant amount of (d7)d18:1 was still converted into (d7)d18:2. This was confirmed *in vitro*, by adding either the free LCB (d7)d18:1 or the *N*-acylated d18:1/6:0 to cell-free extract of FADS3-expressing HEK cells. Both the free and the *N*-acylated LCBs were effectively converted into their dienic forms (Fig. 2*F*), indicating that FADS3 is capable of metabolizing both free and *N*-acylated LCBs.

**Figure 3.**
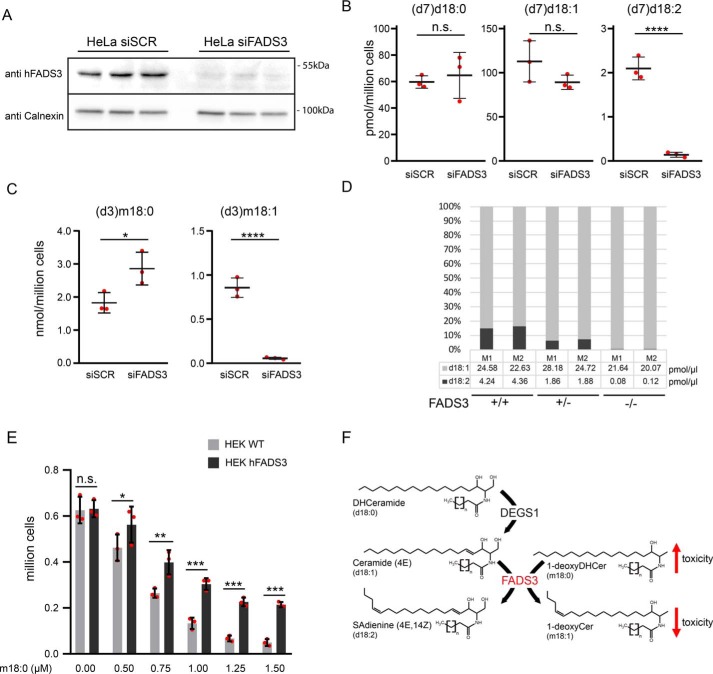
**FADS3 is required for Δ14Z LCB desaturation.** *A*, HeLa cells transfected with siFADS3 or siSCR. The silencing of hFADS3 was confirmed by Western blotting with a polyclonal antibody against FADS3. Calnexin was used as a loading control (*n* = 3). *B–D*, sphingoid base profiling of cells and plasma. *B*, HeLa cells were transfected with siSCR and siFADS3 for 72 h and subsequently cultured for 24 h in the presence of 2 μm (d7)d18:0 and (d3)m18:0. The formation of (d7)d18:2 was significantly decreased in the siFADS3-treated cells. No difference was seen for the precursors (d7)d18:0 and (d7)d18:1. *C*, the conversion of (d3)m18:0 into (d3)m18:1 was also reduced in siFADS3-treated cells compared with controls (siSCR). Data are shown as mean ± S.D. (*error bars*), unpaired *t* test; *, *p* < 0.05; ***, *p* < 0.001. *D*, d18:2 was absent in plasma of the two FADS(−/−) mice (M1/M2), and about 50% in the FADS(+/−) mice compared with WT. Total d18:1 levels were not different. *E*, WT and FADS3-overexpressing HEK293 cells were plated at low density (150,000 cells/well of a 24-well plate) and supplemented with increasing concentrations of m18:0 (0–1.5 μm). After 48 h, the number of attached cells was significantly higher in FADS3-overexpressing cells than in WT. Data are shown as mean ± S.D. (*n* = 3) and compared by two-way analysis of variance with Bonferroni's correction *, *p* < 0.05; **, *p* < 0.01; ***, *p* < 0.001; *n.s.*, not significant. *F*, interplay between the two LCB desaturases DEGS1 and FADS3. DEGS1 introduces the Δ4E DB into d18:0, forming d18:1, whereas FADS3 introduces the Δ14Z DB into d18:1 and m18:0.

For further confirmation, we performed the reverse experiments by knocking down endogenous FADS3 in HeLa cells, which had a higher endogenous FADS3 expression than HEK293 cells. Transfection with FADS3 siRNA (siFADS3) abolished FADS3 expression almost completely compared with cells transfected with control siRNA (siSCR) (Fig. 3*A*). LCB profiling revealed that almost no isotope-labeled (d7)d18:2 was formed in the absence of FADS3, whereas in siSCR-treated cells, a significant conversion of (d7)d18:0 into (d7)d18:1 and finally into (d7)d18:2 was observed (Fig. 3*B*). Similarly, the conversion of isotope-labeled (d3)m18:0 into (d3)m18:1 was mostly abolished in siFADS3-treated cells (Fig. 3*C* and Fig. S4).

Finally, we analyzed the LCB profile in plasma of FADS3-deficient mice (14). No d18:2 was detected in plasma of FADS^−/−^ mice, whereas FADS^+/−^ mice had about 50% of the WT levels (Fig. 3*D*).

To see whether the Δ14Z DB has biological relevance, we cultured WT and FADS3-overexpressing cells with increasing amounts of m18:0 and quantified the number of surviving cells after 48 h. HEK293 cells overexpressing FADS3 were significantly more resistant to m18:0 toxicity than WT cells (Fig. 3*E*).

As d18:2 plasma levels were higher in females, we compared the FADS3 tissue expression between males and females using the public gene expression data from the GTEx portal (https://gtexportal.org).^3^ Overall, the highest FADS3 expression was seen for peripheral nerve, aorta, and adipose tissue. The majority of tissues showed higher FADS3 expression in females. The most pronounced difference was seen for adipose tissues, with 194.6 transcripts per million (TPM) in females *versus* 166.7 TPM in males.

## Discussion

Sphingadienine (d18:2) is an atypical sphingoid base that contains two DBs, a Δ4E and a Δ14Z (3, 15). Whereas the Δ4E DB is introduced by DEGS1 during SL *de novo* synthesis, the enzyme introducing the Δ14Z DB was not known. However, in a gender-, age-, and BMI-matched cohort, we identified d18:2 as the second most abundant LCB (Fig. 1*A*) and significantly higher in females than in males. This confirms data from two recent epidemiologic studies, which also report gender-specific difference in d18:2-based SLs (16, 17).

Although, d18:2 was first described in the late 1960s (3), the responsible enzyme forming the Δ14Z double was not known. Based on genetic association studies in combination with *in vivo* and *in vitro* experiments, we identified FADS3 as a *bona fide* Δ14Z long-chain base desaturase. Data from the GTEx database revealed generally higher FADS3 expression in females for many tissues, including peripheral nerve and adipose tissue, but also liver, kidney, and muscle. Higher FADS3 expression was also reported for female mice (18) and might explain the gender-specific differences in d18:2 plasma levels.

Although human FADS3 was first cloned in 2000 (19), its function remained elusive. FADS3 is composed of an N-terminal cytochrome *b*_5_-like domain and three histidine motifs at the C-terminal ends, which is characteristic for membrane-bound front end desaturases (20). *In vitro* studies indicated that FADS3 is specific for *trans*-vaccenic acid (*trans*-11–18:1), catalyzing the synthesis of a *trans*-11,*cis*-13-linoleic acid isomer (Δ13 desaturation) (21). Interestingly, an earlier GWAS on genetic determinants of circulating sphingolipids found FADS3 to be strongly associated with SM species containing monounsaturated *N*-acyl chains (22). As d18:1-based SLs with unsaturated acyl chains (*e.g.* SM d18:1/24:1) are isobaric to SLs with saturated fatty acids formed on a d18:2 backbone (*e.g.* SM d18:2/24:0), these associations could also refer to -dienic SM species (Figs. S3 and S5).

FADS3 localizes to human chromosome 11q13 (23), which is known as a cancer hotspot locus (19, 24). It is located in a gene cluster together with FADS1 and FADS2, which were identified previously as Δ-5 and Δ-6 desaturases. FADS3 mRNA expression responds inversely to FADS1 and FADS2 expression levels. In FADS2-deficient mice, FADS3 expression increased 3-fold (25), and reducing FADS1 and FADS2 expression by docosahexaenoic acid and arachidonic acid increases expression of FADS3 (26). FADS3 is spliced, yielding to alternative transcripts, which are conserved in several mammalian and avian species (18, 27). However, it is not yet clear whether these alternative transcripts are catalytically active.

Little is known about the function of d18:2-based SLs. Previously, plasma levels of d18:2 SLs were inversely associated with the incidence of cardiovascular events (8), the body mass index, and the HOMA (homeostatic model assessment) index (16). It serves as a backbone for complex sphingolipids (Fig. 1*C*) and forms -dienic LCB phosphates (SAdienine-1P) (29). Free SAdienes and synthetic analogues exhibit cytotoxic and antiproliferative effects in cancer and noncancer cells (30, 31) and were associated with the inhibition of colon tumorigenesis in mouse models (32, 33). Mitochondrial and cellular pools of d18:2-based ceramides were elevated in embryonic fibroblasts derived from double-knockout BAX-BAK mice and a mouse-derived immortalized iBMK cell line (34). It is noteworthy that the Δ14 DB in d18:2 is in *cis* conformation, introducing a kink into the otherwise straight LCB structure. This might affect the lateral assembly of d18:2-based SLs in biological membranes and could interfere with membrane nanodomain formation and receptor clustering. In the first reports, FADS3 KO mice showed no overt phenotype (14). No significant differences in survival, fertility, or growth rate were seen. However, FADS3 KO mice were not yet tested under challenged metabolic conditions, such as high-fat diet. Genetic variants of FADS3 are associated with familial combined hyperlipidemia in the Mexican population (35), and the FADS3 SNP rs174455 was negatively associated with docosahexaenoic acid levels in red blood cell phospholipids (36).

1-DeoxySLs are an atypical class of toxic sphingolipids associated with the rare inherited neuropathy HSAN1 (37) and macular telangiectasia type 2 (38). In contrast to canonical LCBs, m18:1 contains only a single DB in Δ14Z position but lacks the Δ4E DB (5). Here we showed that FADS3 is also responsible for the conversion of m18:0 into m18:1 (Figs. 2*D* and 3*C* and Figs. S2 and S4). In addition, we showed that FADS3-expressing cells were more resistant to m18:0 toxicity than WT cells (Fig. 3*E*). This supports recent reports indicating that m18:0 based 1-deoxySL species are more associated with toxic effects than their unsaturated forms (39). In that respect, FADS3 might contribute to a physiological detoxification of 1-deoxySLs.

It is not fully clear yet whether FADS3 activity is specific for *N*-acylated LCBs or whether it can also metabolize the free form. After blocking *N*-acylation with FB1, we still observed a conversion of (d7)d18:1 into (d7)d18:2, albeit at reduced levels (Fig. 2*E*). Also *in vitro* assays showed that FADS3 (Fig. 2*F*) is capable in converting both the *N*-acylated and the free LCB. However, as free LCBs are typically minor, the conversion of free LCBs is likely of limited biological relevance.

We recently reported that DEGS1 deficiency causes leukodystrophy and peripheral hypomyelination, which was associated with a pathologically increased formation of saturated dihydroSL species (13) and the formation of an atypical monounsaturated d18:1 isomer. Further analysis showed that this isomer contained a Δ14Z DB but lacked the canonical Δ4E DB. This suggests that FADS3 can also metabolize saturated d18:0, although the atypical d18:1 (Δ14Z) isomer was only detected under conditions with reduced DEGS1 activity.

In conclusion, we identified FADS3 as a Δ14Z LCB desaturase introducing a Δ14Z DB into d18:1- and m18:0-based SLs and likely also other LCB substrates. This illuminates the last obscure step in the SL *de novo* synthesis pathways, as the enzyme that forms d18:2 was not yet identified. The activity of FADS3 seems to be higher in females and relates to gender-specific differences in the plasma sphingolipidome. In addition, we propose that FADS3 could be involved in the detoxification of 1-deoxySLs. However, further studies are required to understand the role of FADS3 under physiological and pathophysiological conditions.

## Experimental procedures

### Approvals

The Colaus study was conducted according to the principles expressed in the Declaration of Helsinki and approved by the Institutional Ethics Committee of the University of Lausanne.

Animal studies were approved by the Cornell University Institutional Animal Care and Use Committee (IACUC protocol 2011-0007).

### Human samples and genome-wide association scan

Two subgroups of age- and BMI-matched females and males (*n* = 329 each) were selected from the CoLaus cohort (9, 40). Genotyping and imputation of the cohort was described previously (41). Briefly, individuals with genotyping inconsistencies or with genotyping efficiency below 90% were removed. SNPs with genotyping efficiency below 70% or with Hardy–Weinberg *p* values smaller than 1E−7 were removed. Duplicate individuals and first and second degree relatives were identified by computing genomic identity-by-descent coefficients, using PLINK (42). Imputation was performed using the method of Marchini *et al.* (43), using IMPUTE version 0.2.0, CEU haplotypes, and the fine scale recombination map from HapMap release 21. Given the non-Gaussian distribution of the examined LCBs, inverse normal quantile transformation was applied, regressing out age, sex, and the first four ancestry principal components. The residual trait has been rescaled to have zero mean and unit variance. Linear regression analysis has been performed for 2,557,249 imputed genetic markers as explanatory variables. The probabilistic genotypes were converted to allele dosages.

### Cells and cell culture

HEK293 and HeLa cells (American Type Culture Collection) were cultured in Dulbecco's modified Eagle's medium (Thermo Fisher Scientific) with 10% fetal bovine serum and 1% penicillin/streptomycin (37 °C in 5% CO_2_). Transfection was done using Lipofectamine 3000 (Thermo Fisher Scientific). Transfected cells were cultured under Blasticidin (Thermo Fisher Scientific) for selection. hFADS3 and control siRNA was obtained from Origene and transfected with RNAiMAX (Thermo Fisher Scientific) according to the manufacturer's protocol.

### Immunohistochemistry

HeLa cells were transfected and grown for 24 h on 15-mm coverslips, fixed in 4% paraformaldehyde (30 min, room temperature), and washed in PBS. Cells were blocked for 2 h in PBS, 5% BSA, 1% normal goat serum, and 0.25% Triton X-100) and incubated with the primary antibodies overnight, and anti-calnexin antibody (Sigma, C4731) and anti-V5 antibody (Bio-Rad MCA1360) were used. Secondary antibodies (1:2000, Invitrogen) and 4′,6-diamidino-2-phenylindole (Invitrogen) was added for 4 h. Cells were mounted on glass slides with ProLong Diamond Antifade Mountant (Thermo Fisher Scientific). Pictures were acquired on a confocal laser-scanning microscope (Leica SP8, Leica Microsystems, Wetzlar, Germany, HC PL APO CS2 ×63 numeric aperture, 1.4 oil) at 90.4 nm × 90.4 nm × 300 nm (*x* × *y* × *z*) resolution and analyzed using the Fiji image-processing package (44).

### Protein detection

Protein isolation and Western blotting were carried out as described elsewhere (13).

### Cloning

FADS1 (NM_013402.4), FADS2 (NM_004265.3), and FADS3 (XM_011545023.2) were amplified from a commercially available cDNA library using the following primers: FADS1 Forward, caccatgggaacgcgcgctgcgagg; FADS1 Reverse, ttggtgaagataggcatctagccag; FADS2 Forward, caccatggggaagggagggaaccag; FADS2 Reverse, tttgtgaaggtaggcgtccag; FADS3 Forward, caccatgggcggcgtcggggagcc; FADS3 Reverse, ctgatggaggtaggcgtccagatg.

The PCR product was cloned into the pcDNA3.1 V5-His-TOPO vector (Thermo Fisher Scientific) and verified using Sanger sequencing. Myc-DDK–tagged mouse FADS1–3 plasmids were commercially available (Origene).

### Lipidomics analysis

Lipidomics analysis was performed as described previously (13, 28) using the internal standards (100 pmol/ml, Avanti Polar Lipids): (d5) 1-desoxymethylsphinganine (m17:0), dihydroceramide (d18:0:12:0), ceramide (d18:1/12:0), 1-deoxydihydroceramide (m18:0/12:0), 1-deoxyceramide (m18:1/12:0), glucosylceramide (d18:1/8:0), and sphingomyelin (d18:1/12:0).

The plasma LCB profile was analyzed as described previously (10). For quantification, (d5) 1-desoxymethylsphinganine (m17:0) was used as an internal standard (Avanti Polar Lipids).

### Metabolic labeling assay

250,000 Cells were seeded and cultured in 6-well plates to 70–80% confluence. New medium containing isotope-labeled (d3)m18:0, (d7)d18:0, or (d7)d18:1 (Avanti Polar Lipids) was added. After 24 or 48 h, cells were harvested, counted (Beckman Coulter Vi-Cell XR), pelleted (600 × *g* at 4 °C), and stored at −20 °C.

### Activity assay

FADS3 cells were lysed by sonication, and the homogenate was centrifuged at 13,000 × *g* for 5 min. For each condition, 100 μg of lysate in 100 μl of PBS + 1 mm NAD^+^ were incubated with 500 pmol of (d7)d18:1 or d18:1/6:0 for 5 min. The products were measured by LC-MS.

### Statistical analysis

Statistical analysis was performed using GraphPad Prism 8. A *p* value of <0.05 was considered statistically significant.

## Author contributions

G. K., A. v. E., and T. H. conceptualization; G. K., A. v. E., and T. H. data curation; G. K., A. v. E., and T. H. formal analysis; G. K., Z. K., A. v. E., and T. H. supervision; G. K., Z. K., and T. H. validation; G. K., M. A. L., Z. K., J. T. B., H. L., D. P., A. v. E., and T. H. investigation; G. K., Z. K., and T. H. visualization; G. K. and Z. K. methodology; G. K., Z. K., A. v. E., and T. H. writing-original draft; G. K., Z. K., J. T. B., H. L., A. v. E., and T. H. writing-review and editing; Z. K., J. T. B., H. L., D. P., A. v. E., and T. H. resources; A. v. E. and T. H. funding acquisition; A. v. E. and T. H. project administration.

## Supplementary Material

Supporting Information

## Footnotes

3 Please note that the JBC is not responsible for the long-term archiving and maintenance of this site or any other third party hosted site.

## Footnotes

2 The abbreviations used are:

SL: sphingolipid
LCB: long-chain base
siFADS3: FADS3 siRNA
siSCR: scrambled control siRNA
FA: fatty acid
DB: double bond
SM: sphingomyelin(s)
HexCer: hexosylceramide(s)
Cer: ceramide(s)
T2DM: diabetes type 2
BMI: body mass index
ER: endoplasmic reticulum
TPM: transcripts per million.
