# Supplementary material for: FADS3 is a Δ14Z sphingoid base desaturase that contributes to gender differences in the human plasma sphingolipidome
Source: J Biol Chem. 2019 Dec 20;295(7):1889–97. doi: 10.1074/jbc.AC119.011883 (PMC7029104; doi:10.1074/jbc.AC119.011883)
Supplement: Supporting Information [file supp_295_7_1889__index.html]

FADS3 is a delta14Z sphingoid base desaturase that contributes to gender differences to the human plasma sphingolipidome — FADS3 is a delta14Z sphingoid base desaturase — FADS3 is a Δ14Z sphingoid base desaturase that contributes to gender differences in the human plasma sphingolipidome — ACCELERATED COMMUNICATION: Δ14Z sphingoid base desaturase FADS3 — Supporting Information 

# FADS3 is a Δ14Z sphingoid base desaturase that contributes to gender differences in the human plasma sphingolipidome

## Supporting Information

- supporting information - Supporting information in word format
- Supporting Information (to be published online) - Supporting information in pdf
