# Supplementary material for: FADS3 is a Δ14Z sphingoid base desaturase that contributes to gender differences in the human plasma sphingolipidome
Source: J Biol Chem. 2019 Dec 20;295(7):1889–97. doi: 10.1074/jbc.AC119.011883 (PMC7029104; doi:10.1074/jbc.AC119.011883)
Supplement: Supporting Information [file supp_AC119.011883_157090_1_supp_444417_q2tmbf.docx]

**Supportive Information**

FADS3 is a delta14Z sphingoid base desaturase that contributes to gender differences to the human plasma sphingolipidome

**Gergely Karsai^1^, Museer Lone^1^, Zoltan Kutalik^2,3^, J. Thomas Brenna^4^, Hongde Li^5^, Duojia Pan^5^, Arnold von Eckardstein^1^, Thorsten Hornemann^1*^**

Figure S1. Sphingolipid de-novo synthesis and catabolism.

Figure S2. Lipidomics analysis of HEK cells stably expressing hFADS1-3.

Figure S3. Identification of 1-deoxy-ceramide m42:1 isomers.

Figure S4. Lipidomics analysis of siRNA treated HeLA and HEK293 (WT and hFADS3) cells.

Figure S5. Identification of ceramide d42:2 isomers.


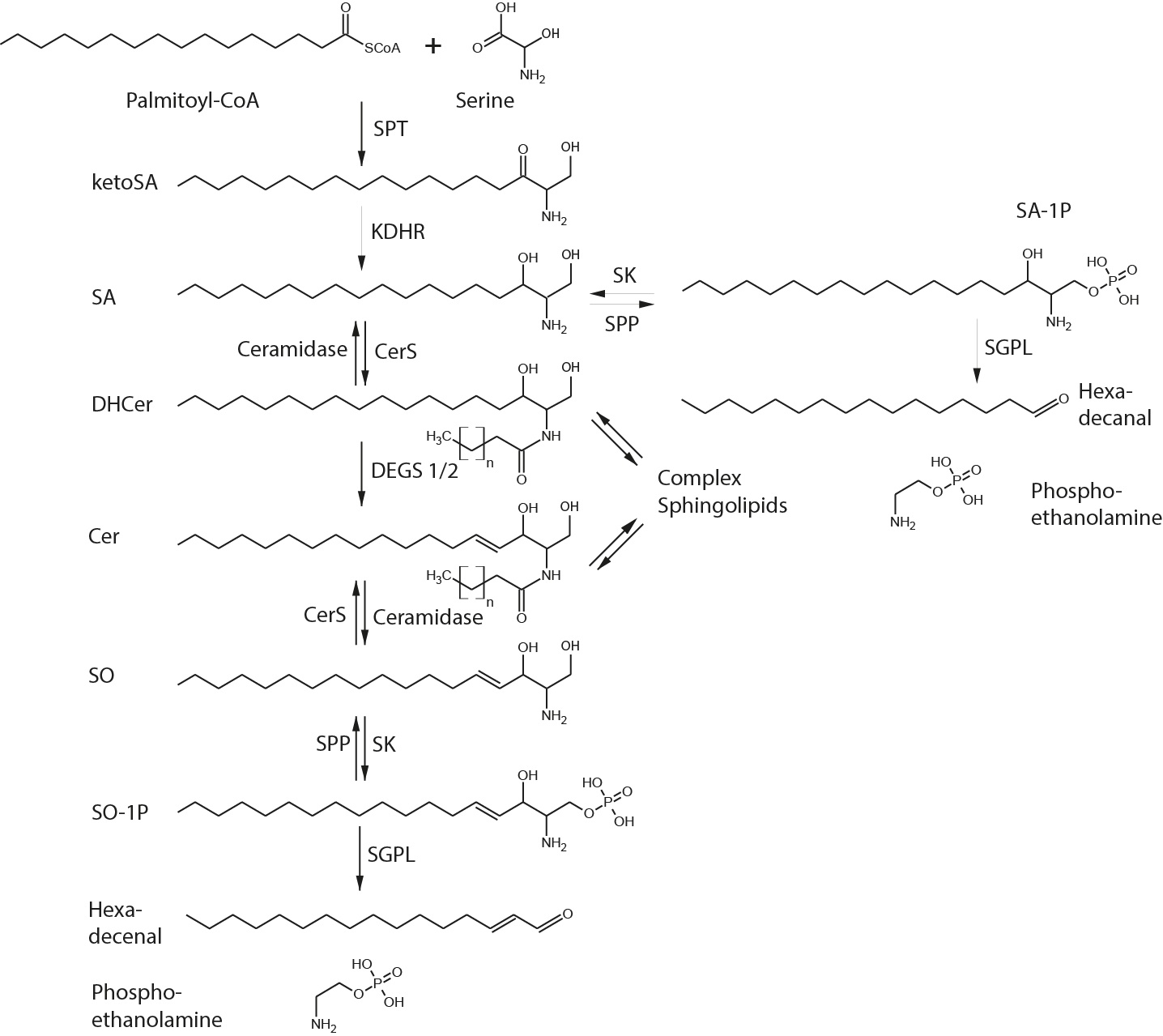


**Figure S1. Sphingolipid de-novo synthesis and catabolism.**

**
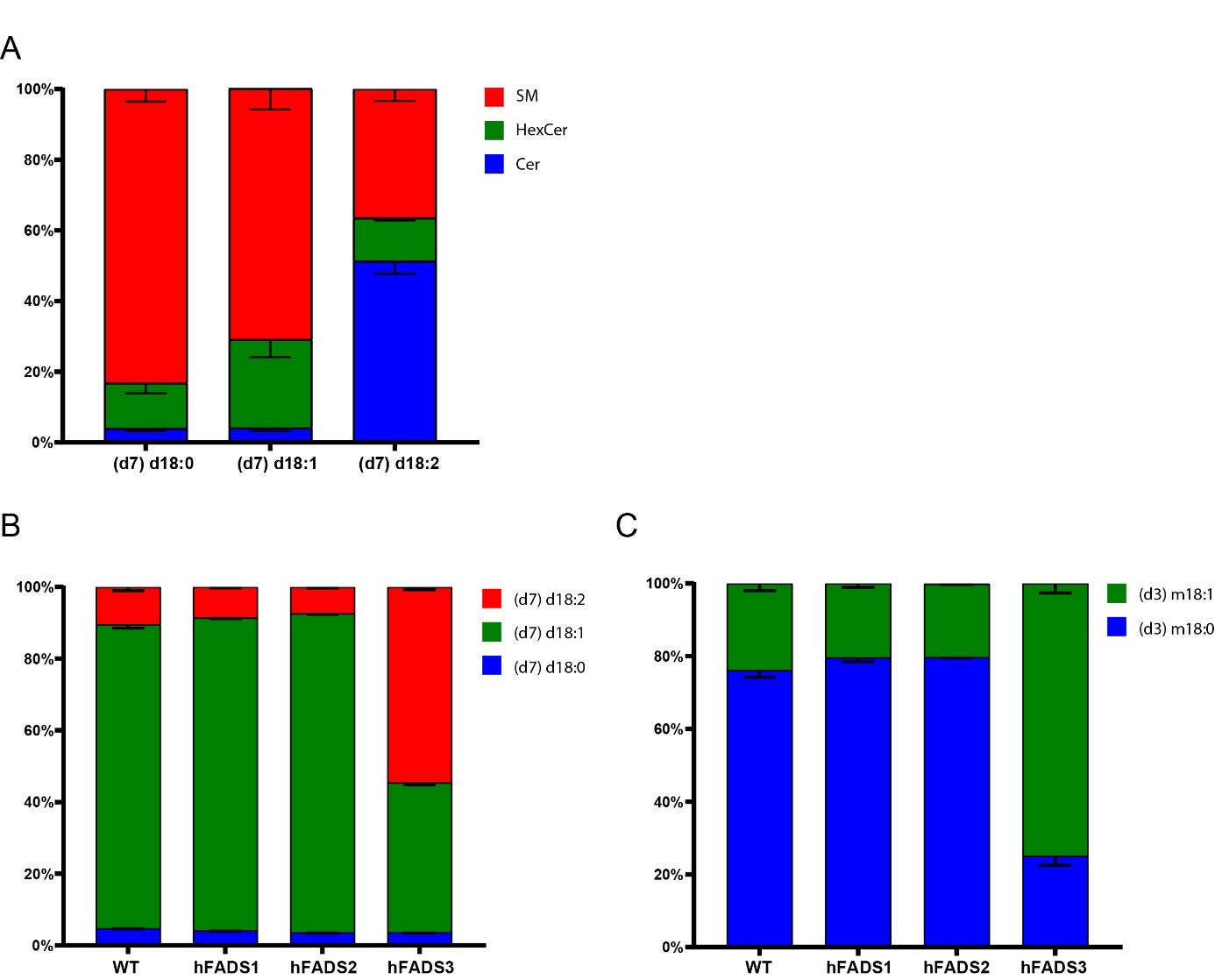
**

**Figure S2. Lipidomics analysis of HEK cells stably expressing hFADS1-3.**

**A-C** Lipidomics analysis of HEK cells treated with (d7)d18:1 (2 µM) or (d3)m18:0 (2 µM) for 48 hours. From each SL class the species with the following N-acyl chains were quantified: C16:0, C18:0, C20:0, C22:0, C24:0 and C24:1. **B** Relative LCB distribution in complex sphingolipids of WT HEK cells. Bars represent the relative proportion of Cer, HexCer and SM that are formed on the respective LCB. **B-C** Relative LCB distribution in HEK cells, stably expressing human (h)FADS1, 2 and 3. **B** The relative distribution of isotope labelled (d7) LCBs was calculated from the sum of Ceramides, HexCers and SMs with the N-acyl chains C16:0, C18:0, C20:0, C22:0, C24:0 and C24:1. **C** The relative distribution of isotope labelled (d3) LCBs was calculated from the sum of 1-deoxy-dihydroceramides, 1-deoxy-ceramides and 1-deoxy-ceramides with m18:1 backbone. Data are shown as means±SD, n=3.
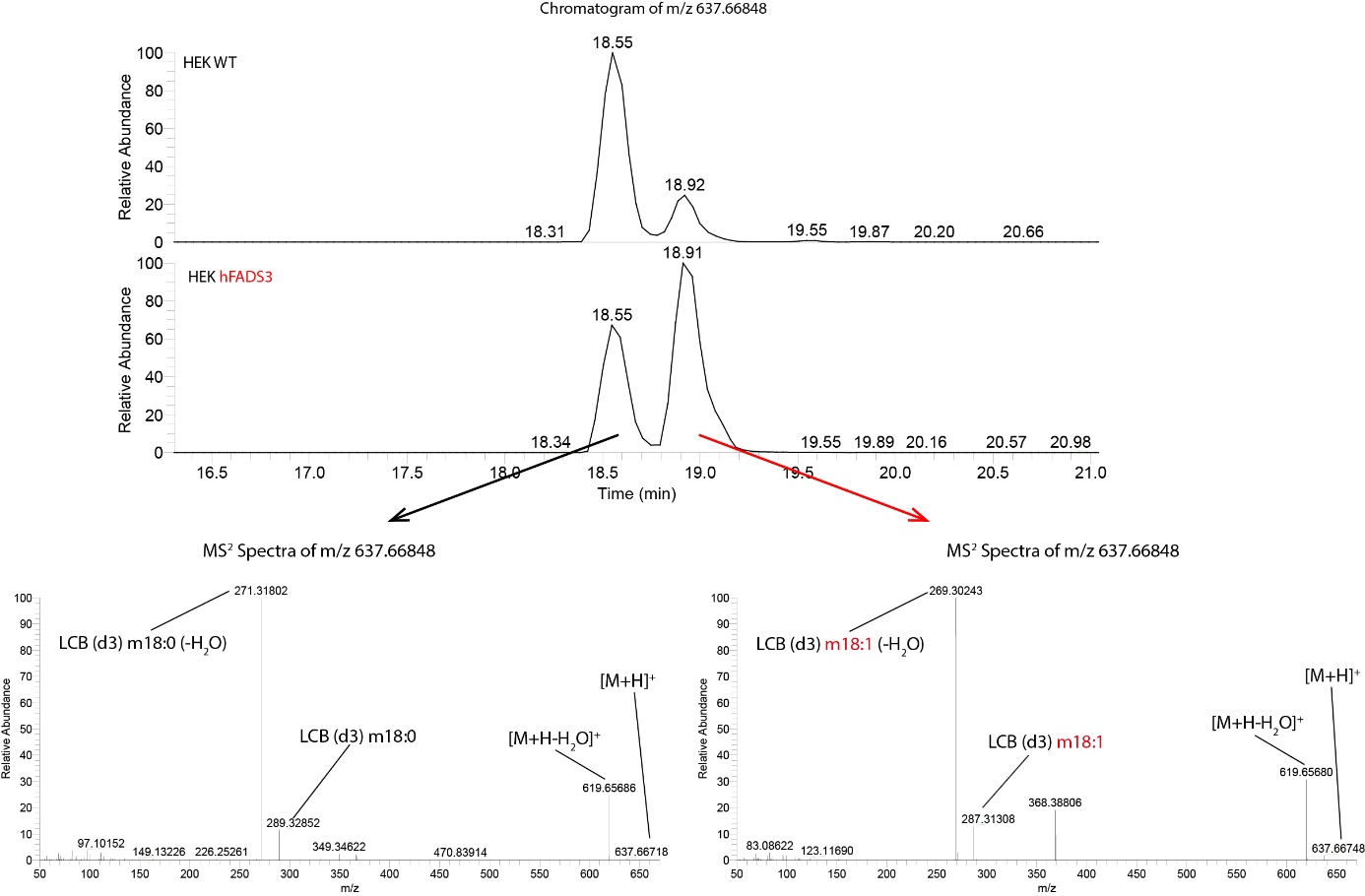
**Figure S3. Identification of (d3) 1-deoxy-ceramide m42:1 isomers.**

Chromatogram of isotope labelled (d3)m42:1 (m/z 637.66484) in positive ion mode of HEK WT and hFADS3 overexpressing cells supplemented with (d3)m18:0. MS^2^ fragment spectrum reveals that the first peak (RT 18:55 min) is (d3)m18:0/24:1, while the second peak (RT 18:91 min) is (d3)m18:1/24:0 due to the LCB fragment.

**
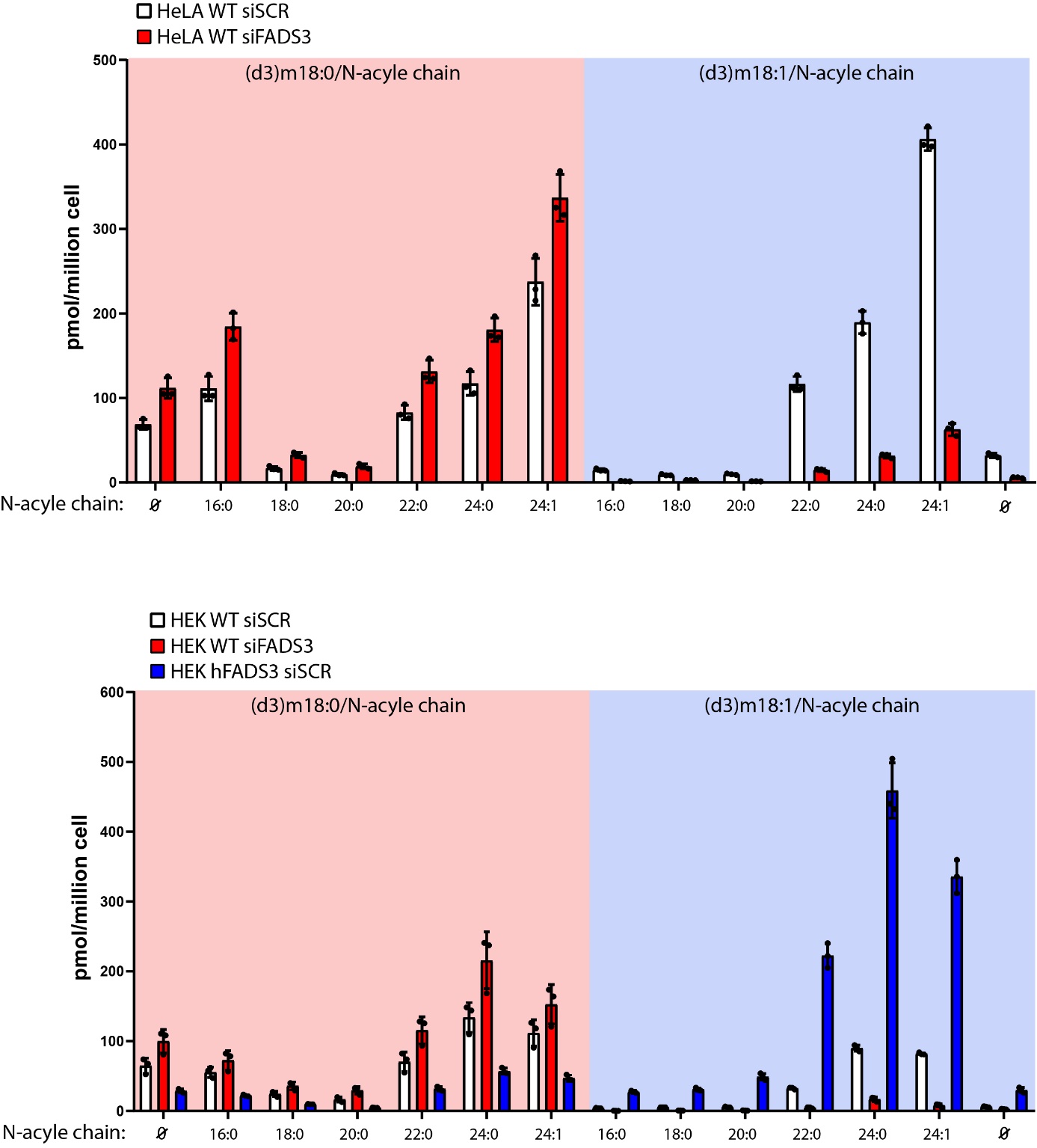
**

**Figure S4. Lipidomics analysis of siRNA treated HeLA and HEK293 (WT and hFADS3) cells.**

Cells treated with siRNA according to experimental procedures and subsequently treated with (d3)m18:0 (2 µM) for 24 hours. Free bases are represented with N-acyl chain: ∅. Data are shown as means±SD, n=3.


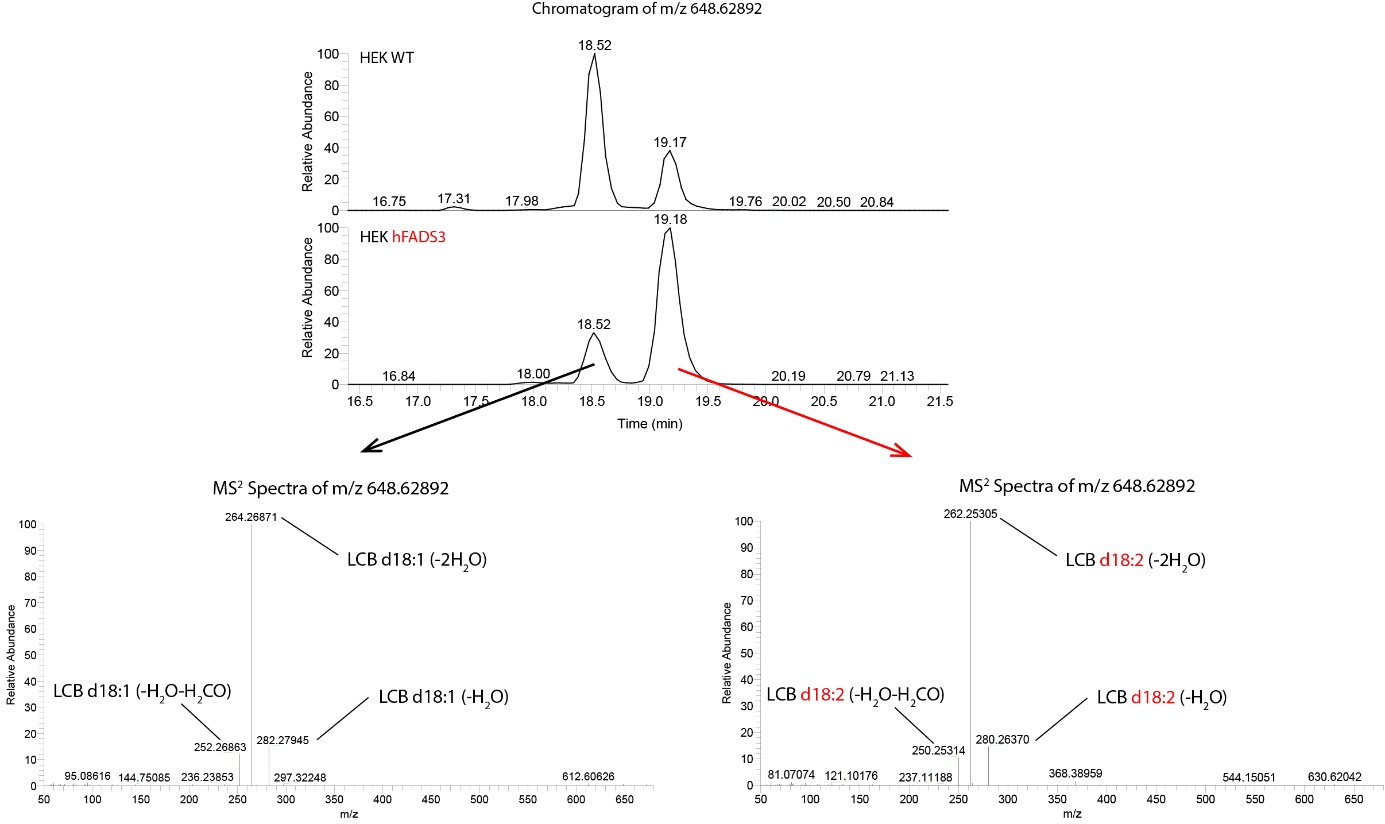


**Figure S5. Identification of ceramide d42:2 isomers.**

Chromatogram of d42:2 (m/z 648.62892) in positive ion mode of HEK WT and hFADS3 overexpressing cells. MS^2^ fragment spectrum reveals that the first peak (RT 18:52 min) is d18:1/24:1, while the second peak (RT 19:17 min) is d18:2/24:0 due to the LCB fragment.
