# Supplementary material for: FADS3 is a Δ14Z sphingoid base desaturase that contributes to gender differences in the human plasma sphingolipidome
Source: J Biol Chem. 2019 Dec 20;295(7):1889–97. doi: 10.1074/jbc.AC119.011883 (PMC7029104; doi:10.1074/jbc.AC119.011883)
Supplement: Supporting Information [file supp_AC119.011883_157090_1_supp_444418_q2tmhb.pdf]

Supportive Information

FADS3 is a delta14Z sphingoid base desaturase that contributes to gender differences to the human plasma sphingolipidome

Gergely Karsai[1], Museer Lone[1], Zoltan Kutalik[2,3], J. Thomas Brenna[4], Hongde Li[5], Duojia Pan[5], Arnold von Eckardstein[1], Thorsten Hornemann[1*]

Figure S1. Sphingolipid de-novo synthesis and catabolism.

Figure S2. Lipidomics analysis of HEK cells stably expressing hFADS1-3.

Figure S3. Identification of 1-deoxy-ceramide m42:1 isomers.

Figure S4. Lipidomics analysis of siRNA treated HeLA and HEK293 (WT and hFADS3) cells.

Figure S5. Identification of ceramide d42:2 isomers.

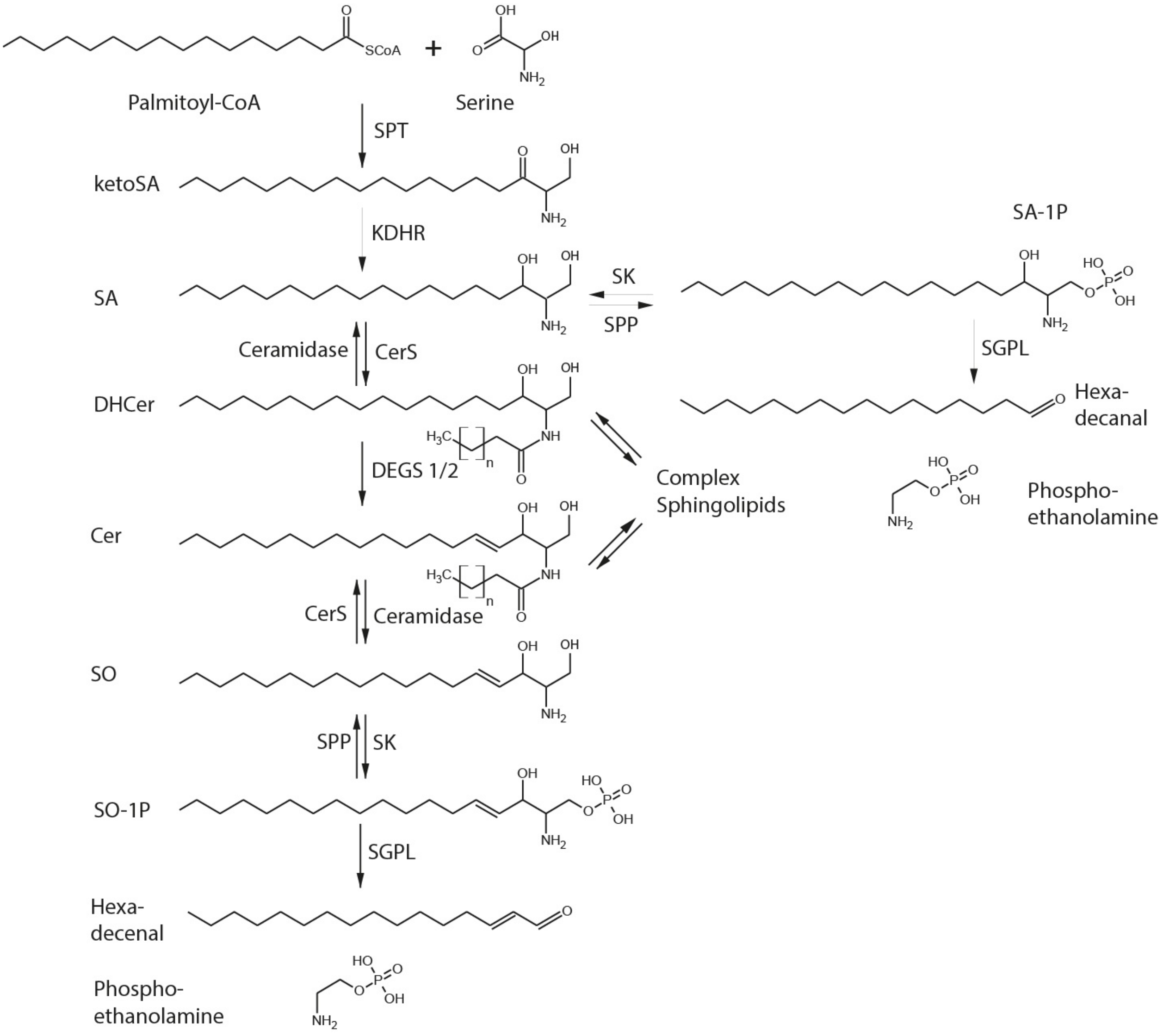

Figure S1. Sphingolipid de-novo synthesis and catabolism.

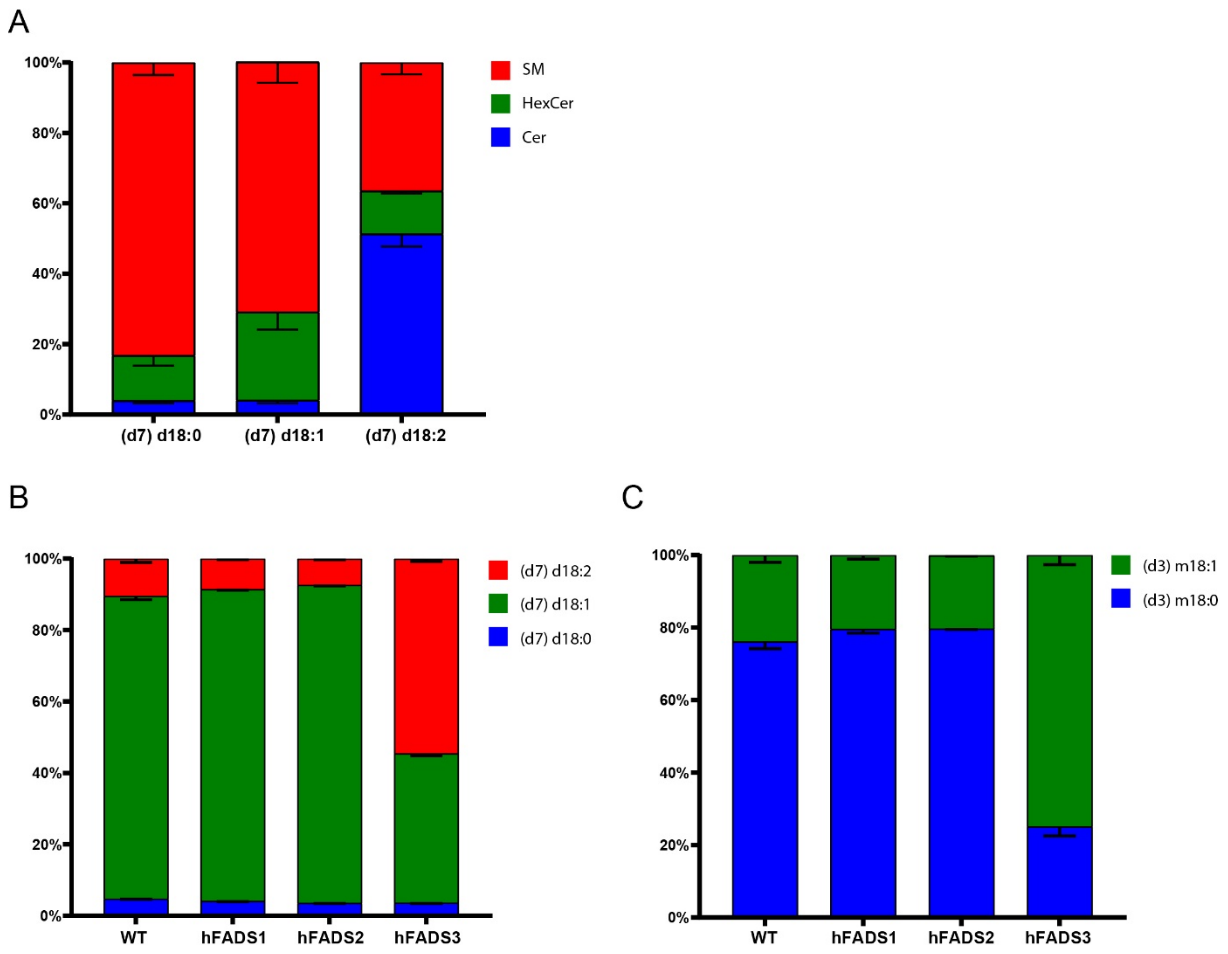

Figure S2. Lipidomics analysis of HEK cells stably expressing hFADS1-3.
A-C Lipidomics analysis of HEK cells treated with (d7)d18:1 (2 µM) or (d3)m18:0 (2 µM) for 48 hours. From each SL class the species with the following N-acyl chains were quantified: C16:0, C18:0, C20:0, C22:0, C24:0 and C24:1. **B** Relative LCB distribution in complex sphingolipids of WT HEK cells. Bars represent the relative proportion of Cer, HexCer and SM that are formed on the respective LCB. **B-C** Relative LCB distribution in HEK cells, stably expressing human (h)FADS1, 2 and 3. **B** The relative distribution of isotope labelled (d7) LCBs was calculated from the sum of Ceramides, HexCers and SMs with the N-acyl chains C16:0, C18:0, C20:0, C22:0, C24:0 and C24:1. **C** The relative distribution of isotope labelled (d3) LCBs was calculated from the sum of 1-deoxy-dihydroceramides, 1-deoxy-ceramides and 1-deoxy-ceramides with m18:1 backbone. Data are shown as means±SD, n=3.

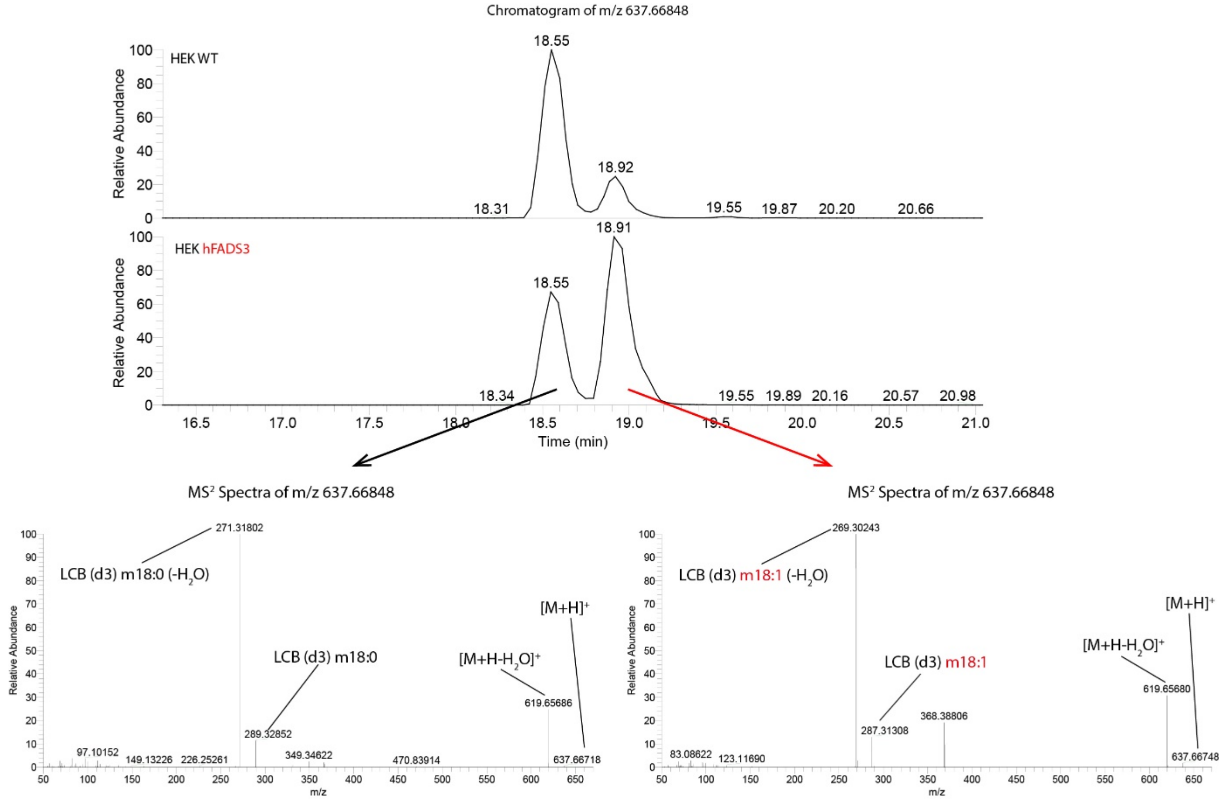

Figure S3. Identification of (d3) 1-deoxy-ceramide m42:1 isomers.
Chromatogram of isotope labelled (d3)m42:1 (m/z 637.66484) in positive ion mode of HEK WT and hFADS3 overexpressing cells supplemented with (d3)m18:0. MS^2 fragment spectrum reveals that the first peak (RT 18:55 min) is (d3)m18:0/24:1, while the second peak (RT 18:91 min) is (d3)m18:1/24:0 due to the LCB fragment.

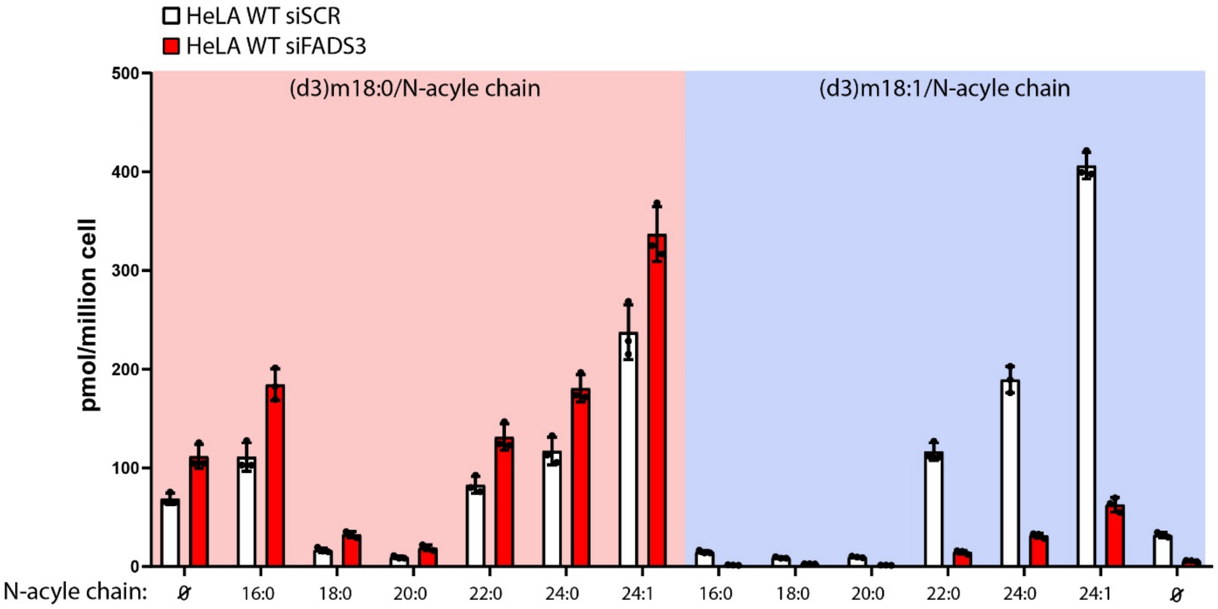

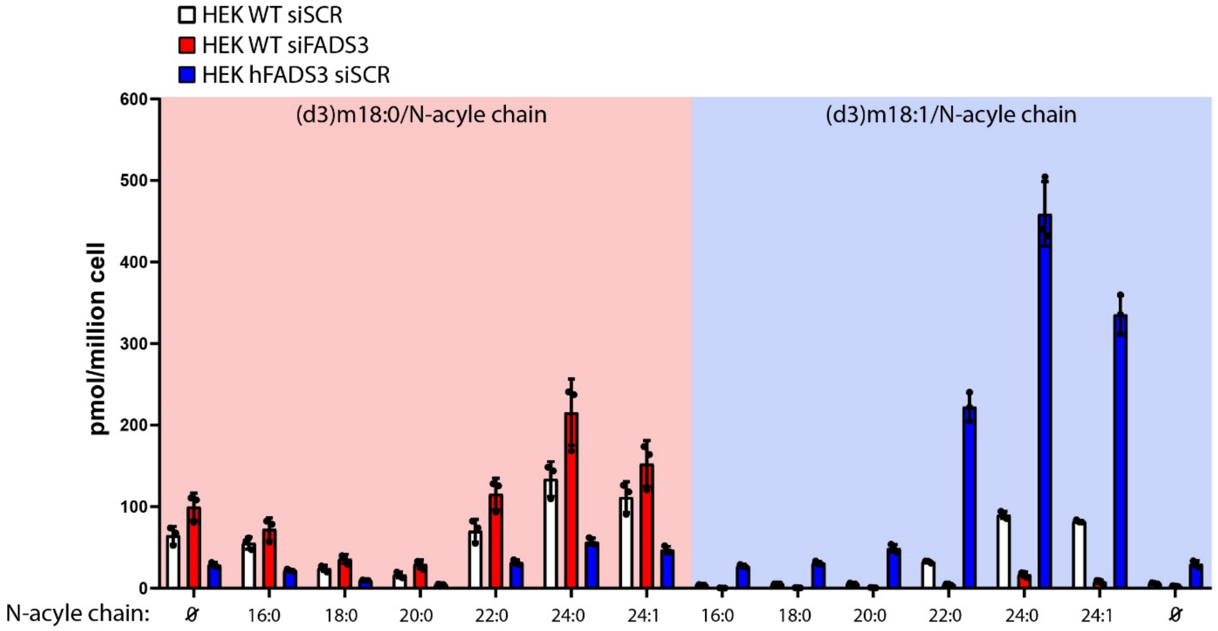

Figure S4. Lipidomics analysis of siRNA treated HeLA and HEK293 (WT and hFADS3) cells. Cells treated with siRNA according to experimental procedures and subsequently treated with (d3)m18:0 (2 µM) for 24 hours. Free bases are represented with N-acyl chain: Ø. Data are shown as means±SD, n=3.

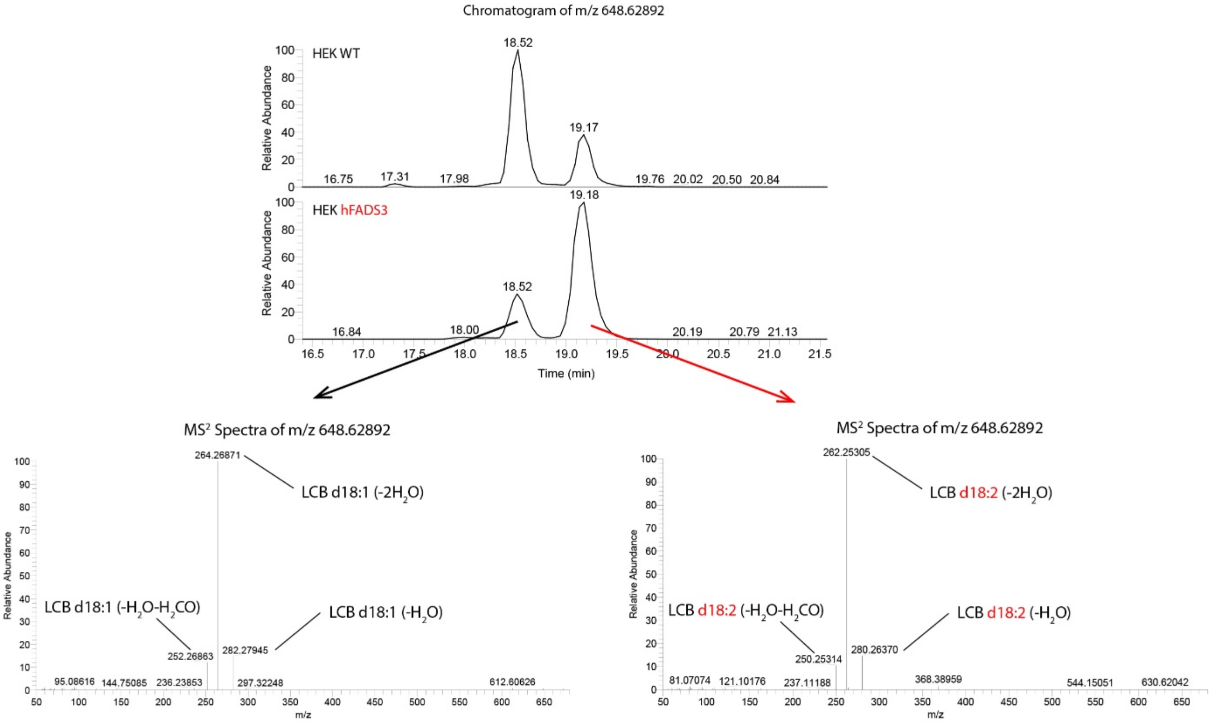

Figure S5. Identification of ceramide d42:2 isomers.

Chromatogram of d42:2 (m/z 648.62892) in positive ion mode of HEK WT and hFADS3 overexpressing cells. MS^2 fragment spectrum reveals that the first peak (RT 18:52 min) is d18:1/24:1, while the second peak (RT 19:17 min) is d18:2/24:0 due to the LCB fragment.
